# An unstructured immersed finite element method for nonlinear solid mechanics

**DOI:** 10.1186/s40323-016-0077-5

**Published:** 2016-07-22

**Authors:** Thomas Rüberg, Fehmi Cirak, José Manuel García Aznar

**Affiliations:** 1 Multiscale in Mechanical and Biological Engineering (M2BE), University of Zaragoza, María de Luna 3, 50018 Zaragoza, Spain; 2 Institut für Baumechanik, TU Graz, Technikerstraße 4, 8010 Graz, Austria; 3 Department of Engineering, University of Cambridge, Trumpington Street, Cambridge, CB2 1PZ UK

**Keywords:** Immersed finite elements, Nonlinear solid mechanics, Nitsche’s method, CSG modelling, Cut-element stabilisation, Implicit geometry

## Abstract

We present an immersed finite element technique for boundary-value and interface problems from nonlinear solid mechanics. Its key features are the implicit representation of domain boundaries and interfaces, the use of Nitsche’s method for the incorporation of boundary conditions, accurate numerical integration based on marching tetrahedrons and cut-element stabilisation by means of extrapolation. For discretisation structured and unstructured background meshes with Lagrange basis functions are considered. We show numerically and analytically that the introduced cut-element stabilisation technique provides an effective bound on the size of the Nitsche parameters and, in turn, leads to well-conditioned system matrices. In addition, we introduce a novel approach for representing and analysing geometries with sharp features (edges and corners) using an implicit geometry representation. This allows the computation of typical engineering parts composed of solid primitives without the need of boundary-fitted meshes.

## Background

Conventional finite element methods (FEM) are an irreplaceable tool for the numerical analysis of a variety of physical and engineering problems. They rely on a conforming mesh which approximately matches the domain boundary and material interfaces. For this reason, mesh generation is an essential part of the workflow in FEM-based analyses [1]. Although the procedure is well-established, often the use of a boundary-conforming mesh can be limiting or even prohibitive. Fluid-structure interaction, large elastic deformations and shape optimisation are some applications where mesh entanglement can cause severe difficulties for conventional FEM.

In the last two decades or so, a number of finite element-based numerical methods have been introduced in order to eliminate the need for boundary-conforming meshes. Here, we restrict ourselves to immersed methods, also known as embedded of fictitious domain methods, that operate with a geometry-independent mesh, in the line of [2–6]. Since the mesh of an immersed domain method does not conform with the boundary of the physical domain, one of these methods’ main difficulties is the application of boundary conditions. Here, we choose Nitsche’s method [7] for the weak enforcement of Dirichlet boundary conditions because it gives optimal convergence rates without incurring major implementation difficulties. Moreover, the use Langrange multipliers together with its numerical intricacies, such as the fulfilment of the LBB-condition [8], are avoided. For alternative approaches, see [4, 9–14] among others.

A major difficulty of non-body-fitted methods is the accurate integration of the arising volume and surface integrals. Here, we make use of a tessellation concept which allows to incorporate standard, Gauß quadrature schemes. In the course of this development, a technique is presented which enables the representation of sharp domain features by performing constructive solid geometry (CSG) modelling directly on the embedding mesh. This approach poses a clear advantage in comparison to the conventional methods of geometry resolution because these sharp features are accurately reproduced and not chamfered even on coarse meshes.

Another pitfall of immersed finite element methods is the loss of numerical stability in cases where the intersection of a shape function support with the physical domain becomes very small. This issue has been successfully addressed in the context of b-spline finite elements [6, 12, 15]. In this work, we build up on this concept of constraining critical degrees of freedom and apply it to Lagrangian basis functions on unstructured meshes. Note that Burman et al. [16] introduced an alternative approach, the so-called *ghost-penalty stabilisation method*, which is based on an augmented bilinear form. Strongly related to stability are the method’s parameters and we show how to choose these parameters in the context of the introduced stabilisation techniques.

The method we present here is based on our previous works [6, 17–19] and related to [2, 3, 16, 20, 21]. Although, as shown in the cited works, the method can be transferred to many physical applications, we focus on the problem class of nonlinear elasticity.

## Weak enforcement of boundary and interface conditions

At first, we present the derivation of the proposed immersed finite element method as applied to boundary value problems from nonlinear solid mechanics. Using a weighted residual technique, we obtain the weak form of the problem and give its linearisation. Similarly, the expressions for material interface problems are subsequently derived.

**Fig. 1:**
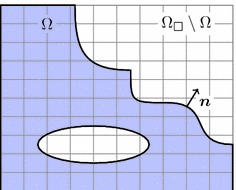
Embedded boundary value problem. Domain $\Omega$ embedded into the domain $\Omega _\square$ with background mesh

### Boundary value problems of nonlinear solid mechanics

Consider the boundary value problem for nonlinear elasticity in the reference domain $\Omega \in \mathbb {R}^{n_d}$, $n_d = 2$ or $n_d=3$ (Fig. 1)

1 $$\begin{aligned} \begin{array}{lllll} \quad -{{\mathrm{Div}}}&{}\,\,\varvec{P}(\varvec{u}) &{}= \varvec{f}\qquad &{}&{}\text {in }\Omega \\ &{}\qquad \varvec{u}&{}= \bar{\varvec{u}} \qquad &{}&{}\text {on }\Gamma _D\\ &{}\quad \varvec{t}(\varvec{u}) &{}= \bar{\varvec{t}} \qquad &{}&{}\text {on }\Gamma _N\,, \end{array} \end{aligned}$$

where $\varvec{u}$ denotes the unknown displacement field, $\varvec{P}$ is the first Piola-Kirchhoff stress tensor, ${{\mathrm{Div}}}$ is the divergence with respect to reference domain coordinates, and $\varvec{t}(\varvec{u}) = \varvec{P}(\varvec{u})\varvec{n}$, with $\varvec{n}$ the outward unit normal vector to $\Omega$, is the boundary traction. The prescribed data are the volume force $\varvec{f}$, the prescribed displacement $\bar{\varvec{u}}$ and the prescribed traction $\bar{\varvec{t}}$. The boundary of $\Omega$, denoted by $\Gamma$, is composed of disjoint sets, the Dirichlet boundary $\Gamma _D$ and the Neumann boundary $\Gamma _N$, where the respective data are given.

In order to construct a weak form of the boundary value problem (1), a weighted residual approach is taken with the test function $\varvec{v}$. In mathematical terms, we operate with the Sobolev space $\varvec{H}^1(\Omega )$, i.e. the vector fields whose components are all in $H^1(\Omega )$, see, among others, [8] for the precise definition. Different from conventional FEM, we do not employ a constrained subspace with essential boundary conditions. The weighted residual method thus becomes:

2 $$\begin{aligned} \text {Find }\varvec{u}\in & {} \varvec{H}^1(\Omega ) \nonumber \\ R(\varvec{u},\varvec{v})= & {} a(\varvec{u},\varvec{v}) - \int _\Omega \varvec{f}\cdot \varvec{v}\mathrm {d}\Omega - \int _{\Gamma _N} \bar{\varvec{t}} \cdot \varvec{v}\mathrm {d}\Gamma - \int _{\Gamma _D} \varvec{t}(u) \cdot \varvec{v}\mathrm {d}\Gamma = 0 \nonumber \\&\forall \varvec{v}\in \varvec{H}^1(\Omega )\,, \end{aligned}$$

with

3 $$\begin{aligned} a(\varvec{u},\varvec{v}) = \int _\Omega \varvec{S}(\varvec{u}) :\dot{\varvec{E}}(\varvec{v}) \mathrm {d}\Omega \,. \end{aligned}$$

Here, $\dot{\varvec{E}}$ denotes the variation of the Euler-Green strain tensor ($\varvec{E} = \tfrac{1}{2}( \varvec{F}^\top \varvec{F} - \varvec{I})$ with the deformation gradient $\varvec{F}$) and $\varvec{S} = \varvec{F}^{-1} \varvec{P}$ the second Piola-Kirchhoff stress tensor [22, 23]. In the applications section, we work with a compressible Neo-Hooke material with given energy density $W(\varvec{E})$ and for this hyperelastic case, the stress tensor becomes

4 $$\begin{aligned} \varvec{S} = \frac{\partial W}{\partial \varvec{E}}\,. \end{aligned}$$

Using a Newton method to solve the nonlinear Eq. (2), the *k* th iteration takes the form

5 $$\begin{aligned} \mathrm {D}R(\varvec{u}^{(k)},\varvec{v})[\Delta \varvec{u}] = - R(\varvec{u}^{(k)}, \varvec{v}) \quad \text {and} \quad \varvec{u}^{(k+1)} = \varvec{u}^{(k)} + \Delta \varvec{u}\,, \end{aligned}$$

where $\mathrm {D}(\cdot )[\Delta \varvec{u}]$ denotes the derivative in direction of the increment $\Delta \varvec{u}$, which reads

6 $$\begin{aligned} \mathrm {D}R(\varvec{u},\varvec{v})[\Delta \varvec{u}] = \mathrm {D}a(\varvec{u},\varvec{v})[\Delta \varvec{u}] - \int _{\Gamma _D} \mathrm {D}\varvec{t}(\varvec{u})[\Delta \varvec{u}] \cdot \varvec{v}\mathrm {d}\Gamma \end{aligned}$$

with

7 $$\begin{aligned} \mathrm {D}a(\varvec{u},\varvec{v})[\Delta \varvec{u}] = \int _\Omega ({{\mathrm{Grad}}}\varvec{v}) :\hat{\varvec{C}}(\varvec{u}) :({{\mathrm{Grad}}}\Delta \varvec{u}) \mathrm {d}\Omega \,. \end{aligned}$$

In this expression, $\hat{\varvec{C}}$ denotes the *effective* elasticity tensor [22]. For simplicity, it is assumed here that the prescribed volume and surface forces, $\varvec{f}$ and $\bar{\varvec{t}}$, are independent of the displacement $\varvec{u}$ (dead load case). If these assumptions do not hold, the directional derivative of $R(\varvec{u},\varvec{v})$ contains the derivatives of the applied force and traction terms. So far, expressions (2) and, consequently, (5) do not take into account the displacement boundary condition $\varvec{u}= \bar{\varvec{u}}$ on $\Gamma _D$. Therefore, the approach initially introduced by Nitsche [7] is adapted here and the following two terms are added to (5)

8 $$\begin{aligned} - \int _{\Gamma _D} \mathrm {D}\varvec{t}(\varvec{u})[\varvec{v}] \cdot (\Delta \varvec{u}- \tilde{\varvec{u}})\mathrm {d}\Gamma \quad \text {and} \quad \gamma \int _{\Gamma _D} (\Delta \varvec{u}- \tilde{\varvec{u}}) \cdot \varvec{v}\mathrm {d}\Gamma \end{aligned}$$

**Fig. 2:**
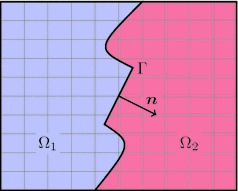
Interface problem. Rectangular domain $\Omega$ composed of two subdomains $\Omega _1$ and $\Omega _2$ with common interface $\Gamma$

with the predictor $\tilde{\varvec{u}} = \bar{\varvec{u}}$ in the first iteration (or the appropriate value in the first iteration of every load step) and $\tilde{\varvec{u}}=\varvec{0}$ otherwise, corresponding to a displacement-controlled Newton method. The scalar $\gamma > 0$, necessary for numerical stability, is discussed in “Numerical stability” section. In summary, the Newton step (5) including Nitsche’s approach to incorporate displacement boundary conditions reads

9 $$\begin{aligned}&\mathrm {D}a(\varvec{u},\varvec{v})[\Delta \varvec{u}] - \int _{\Gamma _D} \mathrm {D}\varvec{t}(\varvec{u})[\Delta \varvec{u}] \cdot \varvec{v}\mathrm {d}\Gamma - \int _{\Gamma _D} \mathrm {D}\varvec{t}(\varvec{u})[\varvec{v}] \cdot \Delta \varvec{u}\mathrm {d}\Gamma \nonumber \\&\quad +\, \gamma \int _{\Gamma _D} \Delta \varvec{u}\cdot \varvec{v}\mathrm {d}\Gamma = -a(\varvec{u},\varvec{v}) + \int _\Omega \varvec{f}\cdot \varvec{v}\mathrm {d}\Omega + \int _{\Gamma _N} \bar{\varvec{t}} \cdot \varvec{v}\mathrm {d}\Gamma \nonumber \\&\quad + \int _{\Gamma _D} \varvec{t}(\varvec{u}) \cdot \varvec{v}\mathrm {d}\Gamma - \int _{\Gamma _D} \mathrm {D}\varvec{t}(\varvec{u})[\varvec{v}] \cdot \tilde{\varvec{u}} \mathrm {d}\Gamma + \gamma \int _{\Gamma _D} \tilde{\varvec{u}} \cdot \varvec{v}\mathrm {d}\Gamma \,, \end{aligned}$$

where the iteration counter has been omitted for sake of legibility. The added terms (8) are zero for the exact solution and therefore the method is consistent by construction. Moreover, for hyperelastic materials expression (9) is symmetric and positive for the right choice of $\gamma$ and non-softening material behaviour, see “Numerical stability” section. In the following, we abbreviate (9) by

10 $$\begin{aligned} A(\varvec{u}; \Delta \varvec{u},\varvec{v}) = \ell (\varvec{u}; \varvec{v})\,. \end{aligned}$$

### Material interfaces

The formalism presented above for the weak incorporation of displacement boundary conditions can be generalised to interface problems, see also [2, 3, 13]. For simplicity, let the reference domain be composed of two subdomains, $\Omega = \Omega _1 \cup \Omega _2$, and let us ignore the Dirichlet boundary conditions on $\partial \Omega$. The treatment of such conditions is here essentially the same as in “Boundary value problems of nonlinear solid mechanics” section. We focus only on the conditions imposed on the material interface $\Gamma = \partial \Omega _1 \cap \partial \Omega _2$, see Fig. 2. In each subdomain $\Omega _i$ the local equilibrium reads

11 $$\begin{aligned} - {{\mathrm{Div}}}\varvec{P}_i(\varvec{u}_i) = \varvec{f}_i \quad \text {in }\Omega _i\,. \end{aligned}$$

Let $\varvec{u}$ denote the compound displacement field, such that $\varvec{u}|_{\Omega _i} = \varvec{u}_i$, and define the compound test function $\varvec{v}$ similarly. Moreover, for any compound function $\varvec{g}$, with $\varvec{g}|_{\Omega _i} = \varvec{g}_i$, the jump across $\Gamma$ is denoted with

12 $$\begin{aligned} \llbracket \varvec{g} \rrbracket = \varvec{g}_1 - \varvec{g}_2\,. \end{aligned}$$

For later use, also a weighted average $\{ \varvec{g} \}$ is defined on $\Gamma$ as

13 $$\begin{aligned} \{ \varvec{g} \} = \beta \varvec{g}_1 + (1-\beta ) \varvec{g}_2\,, \end{aligned}$$

where $0\le \beta \le 1$ is some weighting parameter yet to be discussed. On the interface $\Gamma$ the conditions are

14 $$\begin{aligned} \llbracket \varvec{u} \rrbracket = \varvec{u}^\Gamma \qquad \text {and} \qquad \llbracket \varvec{t}(\varvec{u}) \rrbracket = \varvec{t}^\Gamma \,, \end{aligned}$$

with prescribed jump functions $\varvec{u}^\Gamma$ and $\varvec{t}^\Gamma$. These conditions represent the jump in the solid displacements and the traction equilibrium across the interface. In more complex situations, such as soft interfaces, a cohesive law can be imposed relating the interface traction $\varvec{t}^\Gamma$ to the displacement gap $\llbracket \varvec{u} \rrbracket$, see [24]. Here, we assume that $\varvec{u}^\Gamma$ and $\varvec{t}^\Gamma$ are prescribed and that they are independent of the displacement $\varvec{u}$. Note that in the evaluation of the tractions $\varvec{t}_i$ the unique normal vector $\varvec{n}= \varvec{n}_1 = -\varvec{n}_2$ as shown in Fig. 2 is used, where this choice is arbitrary.

Repetition of the steps as in the single-domain problem above yields the weighted residual method for interface problems

15 $$\begin{aligned} \text {Find }\varvec{u}_i\in & {} \varvec{H}^1(\Omega _i)\quad \, i=1,2, \text {such that}\nonumber \\ R(\varvec{u},\varvec{v})= & {} \sum _{i=1,2} \left( a_i(\varvec{u}_i,\varvec{v}_i) - \int _{\Omega _i} \varvec{f}_i \cdot \varvec{v}_i \mathrm {d}\Omega \right) - \int _\Gamma \llbracket \varvec{t}(\varvec{u}) \cdot \varvec{v} \rrbracket \mathrm {d}\Gamma = 0 \nonumber \\&\forall \varvec{v}_i \in \varvec{H}^1(\Omega _i). \end{aligned}$$

Now the integrand of the interface term is rewritten as follows

16 $$\begin{aligned} \begin{array}{ll} \llbracket \varvec{t}(\varvec{u}) \cdot \varvec{v} \rrbracket &{}= \left[ \beta \varvec{t}_1(\varvec{u}_1) + (1-\beta ) \varvec{t}_2 (\varvec{u}_2) \right] \cdot \llbracket \varvec{v} \rrbracket + [ (1-\beta ) \varvec{v}_1 + \beta \varvec{v}_2 ] \cdot \llbracket \varvec{t}(\varvec{u}) \rrbracket \\ &{}= \{ \varvec{t}(\varvec{u}) \} \llbracket \varvec{v} \rrbracket + [(1-\beta ) \varvec{v}_1 + \beta \varvec{v}_2 ] \cdot \varvec{t}^\Gamma \end{array} \end{aligned}$$

employing the average term (13) and the interface conditions (14)$_2$. Using a Newton method to solve the nonlinear problem (15) with (16) requires the directional derivative

17 $$\begin{aligned} \mathrm {D}R(\varvec{u},\varvec{v})[\Delta \varvec{u}] = \sum _{i=1,2} \mathrm {D}a(\varvec{u}_i,\varvec{v}_i)[\Delta \varvec{u}_i] - \int _\Gamma \{ \mathrm {D}\varvec{t}(\varvec{u}) [\Delta u ] \} \cdot \llbracket \varvec{v} \rrbracket \mathrm {d}\Gamma \,. \end{aligned}$$

The interface condition (14)$_1$ is now incorporated by adding terms akin to (8), namely

18 $$\begin{aligned} - \int _\Gamma \{ \mathrm {D}\varvec{t}(\varvec{u}) [v] \} \cdot (\llbracket \Delta \varvec{u} \rrbracket - \tilde{\varvec{u}}^\Gamma ) \mathrm {d}\Gamma \quad \text {and} \quad \gamma \int _\Gamma (\llbracket \Delta \varvec{u} \rrbracket - \tilde{\varvec{u}}^\Gamma ) \cdot \llbracket \varvec{v} \rrbracket \mathrm {d}\Gamma \,, \end{aligned}$$

to expression (17). Again, the parameter $\gamma >0$ is yet to be discussed in the Appendix A and we use the predictor $\tilde{\varvec{u}}^\Gamma = \varvec{u}^\Gamma$ in the first iteration and zero afterwards. In summary, a step in a Newton iteration to solve the coupled interface problem reads

19 $$\begin{aligned}&\sum _{i=1,2} \mathrm {D}a_i(\varvec{u}_i,\varvec{v}_i) [\Delta \varvec{u}_i] - \int _\Gamma \{ \mathrm {D}\varvec{t}(\varvec{u})[\Delta \varvec{u}] \} \cdot \llbracket \varvec{v} \rrbracket \mathrm {d}\Gamma - \int _\Gamma \{ \mathrm {D}\varvec{t}(\varvec{u})[\varvec{v}] \} \cdot \llbracket \Delta \varvec{u} \rrbracket \mathrm {d}\Gamma \nonumber \\&\quad +\, \gamma \int _\Gamma \llbracket \Delta \varvec{u} \rrbracket \cdot \llbracket \varvec{v} \rrbracket \mathrm {d}\Gamma = \sum _{i=1,2} \left( \int _{\Omega _i} \varvec{f}_i \cdot \varvec{v}_i \mathrm {d}\Omega - a_i(\varvec{u}_i,\varvec{v}_i) \right) + \int _\Gamma \{ \varvec{t}(\varvec{u}) \} \cdot \llbracket \varvec{v} \rrbracket \mathrm {d}\Gamma \nonumber \\&\quad - \int _\Gamma \{ \mathrm {D}\varvec{t}(\varvec{u}) [\varvec{v}] \} \cdot \tilde{\varvec{u}}^\Gamma \mathrm {d}\Gamma + \int _\Gamma \left\{ (1-\beta ) \varvec{v}_1 + \beta \varvec{v}_2 \right\} \cdot \varvec{t}^\Gamma \mathrm {d}\Gamma + \gamma \int _\Gamma \tilde{\varvec{u}}^\Gamma \cdot \llbracket \varvec{v} \rrbracket \mathrm {d}\Gamma \,. \end{aligned}$$

Only the first two terms on the right hand side remain in the case of $\varvec{u}^\Gamma =\varvec{0}$ and $\varvec{t}^\Gamma = \varvec{0}$. As before, this expression is represented by the equation $A(\varvec{u}; \Delta \varvec{u}, \varvec{v}) = \ell (\varvec{u}; \varvec{v})$, see (10), and we postpone the discussion of the parameters $\beta$ and $\gamma$ to Appendix A.

## Immersed finite element method

### Finite element discretisation

**Fig. 3:**
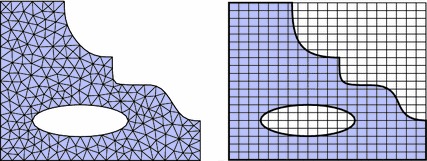
FE discretisation. Geometry-conforming (*left*) and immersed (*right*)

The linearised weighted residual Eqs. (9) and (19) form the basis of a finite element discretisation. To this end, a domain $\Omega _\square$ of a simple shape, typically rectangular, is defined such that it fully contains the reference domain $\Omega$. The following finite element discretisation is based on a triangulation of $\Omega _\square$ instead of a geometry-conforming mesh of $\Omega$ itself (see Fig. 3). We use piece-wise polynomial basis functions $\varphi _I(\varvec{x})$ and write for the approximated displacement field

20 $$\begin{aligned} \varvec{u}^h(\varvec{x}) = \sum _I \varvec{u}_I \varphi _I(\varvec{x})\,. \end{aligned}$$

There is no constraint on the chosen finite element space, but if the surface $\Gamma$ overlaps with the boundary of the embedding domain (that is if $\Gamma _\square = \Gamma \cap \partial \Omega _\square \ne \emptyset$), it can be more convenient to use an essential treatment of displacement boundary conditions [8] along this boundary. On the other hand, if non-nodal basis functions (like, for instance, higher-order b-splines) are used as the finite element basis, the above presented weak incorporation of the boundary conditions works perfectly well on this boundary part $\Gamma _\square$ too.

Let the support of the basis function $\varphi _I$ be denoted by ${{\mathrm{supp}}}(\varphi _I)$. Now all coefficients $\varvec{u}_I$ from the approximation (20) are discarded a priori if ${{\mathrm{supp}}}(\varphi _I) \cap \Omega = \emptyset$. By $\mathbb {S}$ we denote the set of the indices of the remaining coefficients and thus $\{ \varphi _I \}_{I\in \mathbb {S}}$ forms the full basis of the immersed finite element method. This basis is in general not stable [25] and requires further attention, which is given in “Numerical stability” section. Using the approximation (20), we reach the final system of equations

21 $$\begin{aligned} \varvec{\mathsf {\mathbf {A}}} \varvec{\mathsf {\mathbf {x}}} = \varvec{\mathsf {\mathbf {b}}} \end{aligned}$$

with the matrix and vector coefficients

22 $$\begin{aligned} \varvec{\mathsf {\mathbf {A}}}[I\, n_d + a,J\, n_d + b ]= & {} A(\varvec{u}; \varvec{e}_b \varphi _J, \varvec{e}_a \varphi _I) \nonumber \\ \varvec{\mathsf {\mathbf {x}}}[J\, n_d + b]= & {} (\Delta \varvec{u}_J)\cdot \varvec{e}_b \\ \varvec{\mathsf {\mathbf {b}}}[I\, n_d + a]= & {} \ell (\varvec{u}; \varvec{e}_a \varphi _I )\nonumber \end{aligned}$$

for the zero-based indices $I,J \in \mathbb {S}$, and using the coordinate directions $0 \le a,b < n_d$ ($n_d$ being the spatial dimension of the problem) and Cartesian unit vectors $\varvec{e}_a$. Although this immersed finite element method seemingly leads to the same type of linear system as a conventional, geometry-conforming FEM, there are technical differences which will be discussed in the following: the representation of the boundary or interface $\Gamma$, the quadrature of elements traversed by this boundary, and the stabilisation of the basis for such elements. The choice of the Nitsche parameters $\gamma$ and $\beta$ is analysed in the Appendix A.

Above expressions hold analogously for interface problems. The main difference is that the two fields $\varvec{u}_1$ and $\varvec{u}_2$ are approximated in fashion of (20) independently on the same background mesh of $\Omega _\square$ which encompasses both sub-domains $\Omega _1$ and $\Omega _2$. Consequently, the elements which are traversed by the material interface approximate both fields since the FE shape functions of the entire element are used even though the fields are only defined up to the interface on their respective side of the domain. Using two sets of shape functions on these elements allows us to represent a discontinuous derivative of the FE solution and can thus be compared to the element enrichment of XFEM [11]. A good illustration of this implementation detail can be found in [2].

### Signed distance functions

The weak forms introduced in “Weak enforcement of boundary and interface conditions” section allow us to work with a finite element discretisation which is independent of the geometry, but still the volume and surface integrals, $\int _\Omega (\cdot ) \mathrm {d}\Omega$ and $\int _\Gamma (\cdot ) \mathrm {d}\Gamma$, need geometry information. To this end, we classify the elements (for instance the quadrilaterals in the right picture of Fig. 3) by their location with respect to the physical domain $\Omega$. If $\tau _I$ denotes any such element, we have the three cases:

1. $\tau _I \cap \Omega = \emptyset$, the element is completely outside of $\Omega$ and can be ignored,
2. $\tau _I \cap \Omega = \tau _I$, the element is completely inside and its treatment is straightforward as in any geometry-conforming FEM,
3. $\tau _I \cap \Gamma \ne \emptyset$, the element is traversed by the domain’s boundary and requires special consideration.

Note that elements adjacent to the boundary of the embedding mesh (for instance the left or bottom boundaries in the right picture of Fig. 3) technically fall into the third category, but do not pose any difficulty apart from the identification of the element faces which lie on that boundary.

**Fig. 4:**
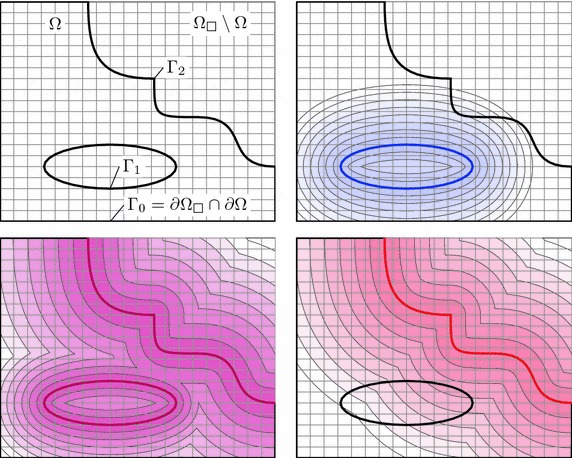
Distance functions of a composite boundary. Geometric constellation (*top left*), distance functions of the boundary parts $\Gamma _1$ (*top right*) and $\Gamma _2$ (*bottom right*), composite distance function ${{\mathrm{dist}}}_\Gamma$ (*bottom left*)

For above classification it is sufficient to have an oriented representation of the surface $\Gamma = \partial \Omega$. Therefore, the surface is either closed or assumed to be extended beyond the boundaries of $\Omega _\square$. Here, we assume that $\Gamma$ is either given analytically or is approximated by means of a surface mesh composed of surface elements $\sigma _J$,

23 $$\begin{aligned} \Gamma \approx \Gamma ^h = \bigcup _J \sigma _J\,. \end{aligned}$$

In order to avoid the tedious task of intersecting volume elements $\tau _I$ with surface elements $\sigma _J$, an *implicit* geometry representation is introduced. Therefore, the signed distance function [26] is used which is defined as

24 $$\begin{aligned} {{\mathrm{dist}}}_{\Gamma }(\varvec{x}) = s(\varvec{x}) \min _{\varvec{y} \in \Gamma } |\varvec{x}- \varvec{y}|\,, \quad \text {with} \quad s(\varvec{x}) = {\left\{ \begin{array}{ll} \phantom {-}1 \quad \text {if }\varvec{x}\in \Omega \\ -1 \quad \text {if }\varvec{x}\notin \Omega \,. \end{array}\right. } \end{aligned}$$

In case of interface problems as introduced in “Material interfaces” section, the above definition of $s(\varvec{x})$ refers to $\Omega _1$ and $\Omega _2$ instead of $\Omega$ and its complement $\Omega _\square \setminus \Omega$. If $\Gamma$ is represented by a mesh $\Gamma ^h$, the signed distance function ${{\mathrm{dist}}}_{\Gamma ^h}$ with respect to this mesh is used instead. Moreover, only a piece-wise polynomial approximation of this function is used

25 $$\begin{aligned} {{\mathrm{dist}}}^h_{\Gamma }(\varvec{x}) = \sum _K d_K \varphi _K(\varvec{x})\quad \text {with } d_K = {{\mathrm{dist}}}_\Gamma (\varvec{x}_K)\,, \end{aligned}$$

where $\varphi _K$ are the nodal finite element shape functions (not necessarily the same as in the approximation (20)) and the coefficients $d_K$ represent the value of the signed distance at the finite element nodes $\varvec{x}_K$.

The representation (23) can be of higher polynomial degree, given by NURBS patches [1, 14, 27] or subdivision surfaces [28, 29]. But the computation of the coefficients $d_K$ in (25) and the quadrature described below are non-trivial tasks if the $\sigma _J$ have a degree higher than linear simplex elements (straight lines in two or flat triangles in three dimensions). In that case, the computation of the distances $d_K$ requires the solution of nonlinear equations, see, for instance, [30]. In the rest of this work, the $\sigma _J$ are always linear $(n_d-1)$-simplex elements. Moreover, once only piece-wise linear elements are used for the surface representation (23), the optimal convergence rate of any higher-order method is impeded by this geometry approximation error, see [8].

Figure 4 shows a two-dimensional example where the boundary is composed of three parts: $\Gamma _0$ is the part of the boundary of $\Omega$ that coincides with the box boundary $\partial \Omega _\square$ and does not require any special attention; $\Gamma _1$ and $\Gamma _2$ are separated parts which are immersed in the background grid. For the computation of the distance function ${{\mathrm{dist}}}_\Gamma$, it is convenient to treat $\Gamma _1$ and $\Gamma _2$ separately as shown in the figure. The final distance function is then composed as the minimal value of these distances,

26 $$\begin{aligned} {{\mathrm{dist}}}_{\Gamma } (\varvec{x}) = \min \left( {{\mathrm{dist}}}_{\Gamma _1}(\varvec{x}), {{\mathrm{dist}}}_{\Gamma _2}(\varvec{x}) \right) . \end{aligned}$$

See also [31] for arithmetic with distance functions. Figure 4 shows the iso-curves of the individual distance functions ${{\mathrm{dist}}}_{\Gamma _i}$ as well as of the composite function ${{\mathrm{dist}}}_{\Gamma }$. The extension of this approach to a larger number of immersed surfaces is straightforward.

Once the function ${{\mathrm{dist}}}_\Gamma$ has been determined, the above classification of volume elements $\tau _I$ is carried out by means of the nodal values $d_K$ of the distance function: if all $d_K$ of the element $\tau _I$ are strictly positive (negative), the element is inside (outside) of the domain. If a change in sign of the $d_K$ occurs, $\tau _I$ is traversed by the immersed boundary $\Gamma$.

**Fig. 5:**
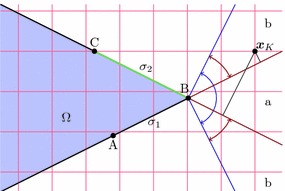
Signed distance computation in the region of an acute corner. The grid point $\varvec{x}_K$ has the vertex B as closest point on the surface, but it lies on opposing sides with respect to the adjacent surface elements $\sigma _1$ and $\sigma _2$; based on the larger distance to the tangent planes of the surface elements $\sigma _i$, the element $\sigma _2$ is chosen to determine the outside position of the point $\varvec{x}_K$

It remains to outline how the coefficients $d_K$ for a given surface are computed. In case of an analytic surface representation by an implicit function, these coefficients are calculated directly. In case of an immersed surface mesh, one needs to find the surface element $\sigma _K^*$ which contains the point $\varvec{x}_K^*$ closest to $\varvec{x}_K$, see for instance [32] for such basic primitive tests as the closest point on a triangle to a point. With the knowledge of the closest element $\sigma _K^*$, it can be decided if $\varvec{x}_K$ lies on the positive or the negative side of this element in order to determine the sign $s(\varvec{x}_K)$ as defined in (24). This decision is based on the premise that the surface mesh is well oriented. Note that, when the closest point falls on an edge or a vertex, ambiguities can arise for the decision if a point is inside or outside the surface mesh [26], see the case shown in Fig. 5.

At the acute corner in the figure, the region of points whose closest point is the vertex B, is delimited by the outer cone. For all points in this cone, $\sigma _1$ and $\sigma _2$ are possible choices as closest surface element. The cone contains the region ‘a’ in which the points are all outside with respect to both elements. The points in region ’b’ are outside with respect to one of the possible closest surface elements and inside with respect to the other. Hence, for this region the mentioned ambiguity can occur. One solution to this problem is to introduce angle-weighted vertex normal vectors [26], but this requires extra data structures. Here we choose the simpler approach shown in Fig. 5: the point $\varvec{x}_K$ has a larger distance to the extension plane of $\sigma _2$ than to the extension plane of $\sigma _1$. This distance is given by the inner product of the element normal vector and the distance vector between the considered grid point and the closest surface point (here, B). Choosing the element with a larger value of this distance resolves the ambiguity. The method is also used in three dimensions with the only difference being a larger set of candidates as closest elements.

Finally, we consider the numerical complexity of the distance function computation. If there are $N_\Gamma$ elements in the surface mesh and $N_\Omega$ nodes in the volume mesh, a brute-force approach requires $N_\Gamma \times N_\Omega$ closest point computations. In many cases, this number can be substantially reduced by precomputing a bounding box [32] of the surface $\Gamma ^h$ and assigning a default value for the $d_K$ of nodes outside of this box, but the essential complexity remains of order $\mathcal {O}(N_\Gamma \times N_\Omega )$. Complexity reduction is possible by generation of a hierarchy of bounding boxes [32] or using so-called *marching* methods, see e.g. [33].

### Constructive solid geometry modelling

**Fig. 6:**
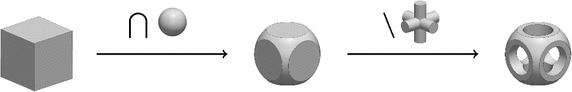
Pipeline of a CSG process. Intersection of a cube with a sphere and removal of three intersecting cylinders

Now, we consider a different approach for integrating finite element analysis with geometric design, similar to the ideas presented in [34]. Specifically, we consider the construction of a three-dimensional geometry by means of CSG, see, for instance, [35, 36]. An example of such a modelling process is given in Fig. 6, where one begins with a cube as a workpiece and performs set operations with other geometric primitives until the desired geometry is obtained. These operations are commonly union $\cup$, intersection $\cap$, subtraction$\setminus$and the set complement $()^\complement$. Based on De Morgan’s laws [36], it suffices to work with the canonical operations intersection and complement, and represent the other two as compositions thereof, more precisely $\mathbb {A} \cup \mathbb {B} = (\mathbb {A}^\complement \cap \mathbb {B}^\complement )^\complement$ and $\mathbb {A}{\setminus }\mathbb {B} = \mathbb {A} \cap \mathbb {B}^\complement$.

The conventional finite element approach is to work through such a CSG pipeline, export a geometry representation and use a mesh generation software to create a body-fitted volume mesh for the numerical analysis. The direct modification for an immersed finite element method is to export a surface representation of the geometry and embed this into the mesh by the methods described in “Immersed finite element method” section. Here, a third way is suggested in which the set operations are directly applied to the embedding (non-conforming) volume mesh. As outlined above, it suffice to provide the complement and intersection operations only. The former is trivially achieved: the use of a signed distance function generates an in- and an outside partition of the mesh, reversing these partitions gives the complement. For this reason, all that need be explained is the intersection operation.

**Fig. 7:**
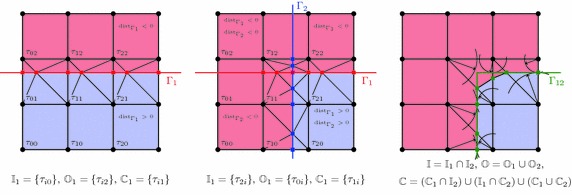
Intersection process. Domain partitioning and element tessellation for a straight boundary $\Gamma _1$ (*left*), resulting constellation for intersection with another line $\Gamma _2$ (*middle*); for comparison see the result of the immersion based on the composite distance function (*right*)

A simple two-dimensional example in Fig. 7 demonstrates the intersection operation: first the intermediate domain $\Omega _1$ is given via the distance function of a straight line $\Gamma _1$, afterwards a second distance function to the line $\Gamma _2$ yields the final domain $\Omega = \{\varvec{x}\in \Omega _\square :{{\mathrm{dist}}}_{\Gamma _1} (\varvec{x})> 0 \text { and } {{\mathrm{dist}}}_{\Gamma _2}(\varvec{x}) > 0 \}$. For sake of clarity, let us discuss the individual steps in this picture. First the line $\Gamma _1$ is embedded into the shown $3\times 3$-grid which fills out the square domain $\Omega _\square$. The elements $\tau _{i0}$ are strictly inside the intermediate domain $\Omega _1 = \{\varvec{x}\in \Omega _\square :{{\mathrm{dist}}}_{\Gamma _1} (\varvec{x}) > 0 \}$ and form the set $\mathbb {I}_1$. The elements $\tau _{i2}$ are strictly outside and form the set $\mathbb {O}_1$. Now, the remaining elements form the set $\mathbb {C}_1$ and are triangulated such that the embedded boundary is approximated by triangle edges. The squares are first subdivided into two triangles each and then every such triangle is intersected by means of the nodal values of the signed distance function ${{\mathrm{dist}}}_{\Gamma _1}$ [37]. The resulting outcome is the left picture in Fig. 7 where the red square-shaped marks indicate the location of the intersection points.

In the second embedding step, the distance function ${{\mathrm{dist}}}_{\Gamma _2}$ is used which gives rise to the element sets $\mathbb {I}_2$, $\mathbb {O}_2$ and $\mathbb {C}_2$. All elements which belong to the outside are directly assigned to the complementary domain $\Omega _\square {\setminus }\Omega$, that is $\mathbb {O} = \mathbb {O}_1 \cup \mathbb {O}_2$. On the other hand, all elements of $\mathbb {I}_1$, which also belong to $\mathbb {I}_2$, are inside the final domain $\Omega$, hence $\mathbb {I} = \mathbb {I}_1 \cap \mathbb {I}_2 = \{\tau _{20}\}$. Finally, there are the intersection cases. Elements belonging to $\mathbb {C}_1$ and $\mathbb {I}_2$ ($\tau _{21}$) keep their status and sub-division. Elements from $\mathbb {I}_1$ and $\mathbb {C}_2$ ($\tau _{10}$) are subject to the same decomposition methods as $\mathbb {C}_1$. It remains to discuss the situation of the elements which belong to $\mathbb {C}_1 \cap \mathbb {C}_2$; the ones which are intersected by both boundaries, and in our example of Fig. 7 this is the element $\tau _{11}$. In this case, simply the composing triangles are intersected with $\Gamma _2$ as if they were elements of their own. Proper categorisation of these simplex shapes defines the final domain $\Omega$ and its complement $\Omega _\square {\setminus }\Omega$, see the middle picture of Fig. 7.

The advantage of this approach becomes clear when looking at the right picture of Fig. 7. Shown is the result for the same target domain $\Omega$, but first the composition of the individual distance functions ${{\mathrm{dist}}}_{\Gamma _i}$ is computed according to expression (26) and then the element intersections are constructed. Clearly, in the right picture the corner is chamfered whereas in the above outlined approach this geometric feature is preserved. This is the distinctive characteristic of the presented idea: by successively embedding the geometry primitives into the mesh, the sharp features at the primitive intersections are preserved. It is important to remark that the boundaries $\Gamma _i$ have been represented exactly in this example, but this is solely owed to the fact that they are straight lines. In the more general situation of curved boundaries, they are again represented on the finite element mesh by piece-wise linear simplex elements. But, even though these surrogate boundaries do not exactly reproduce the given geometry, the here presented approach still allows to represent corners or edges at the intersection locations of the original primitives which lie inside of the finite elements.

**Fig. 8:**
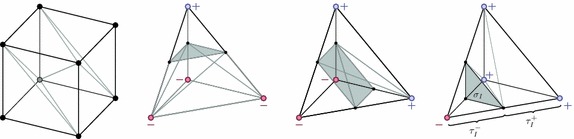
Element subdivision. Decomposition of a hexahedron into six tetrahedrons (*left*) and the fundamental cases of distance function values

The presented method for CSG modelling based on finite element meshes is straightforward to extent to three dimensions. In the plane case outlined so far, the rectangular elements are subdivided into two triangles which themselves are triangulated in order to recover the implicit surface in the form of triangle edges. This approach is akin to a two-dimensional version of marching cubes and in three dimensions we make use of a similar technique. The used three-dimensional element shapes are either tetrahedrons or hexahedrons. Figure 8 shows how a hexahedron is decomposed into six tetrahedrons such that it remains to consider this shape only. Given a tetrahedron with values of the signed distance function at its vertices we can classify the cases shown in the right part of the figure. Based on linear interpolation along the edges the zeros of the distance function are recovered and give rise to two volume tessellations $\tau _I^-$ and $\tau _I^+$ whose common faces form the triangulated surface $\sigma _I$. Using these decompositions, the above outlined intersection operations of two geometry primitives can be carried out analogously in three dimensions.

Alternative approaches for increasing the quality of implicit geometry representations in the vicinity of sharp features (such as edges and corners) exist. In [38] the operations of surface reconstruction by means of marching cubes and the distance function computation are combined in order to generate a so-called directed distance field allowing for a better resolution of surface features. On the other hand, enriched distance functions are presented in [39] where additional edge and vertex descriptors augment the distance geometry representation. Although both approaches are promising concepts in the context of immersed finite element methods, they are not further considered in this work.

We conclude this paragraph by noting that the here used tessellation techniques also help to construct numerical integration schemes for the elements that are traversed by the boundary or interface. The cut elements are general polytopes for which quadrature rules are not easily obtained. There are many techniques that address this problem, such as moment-fitting [40], surface-only integration [41], and adaptive decomposition of the integration region [14, 42]. But since we have a tessellation in simplex shapes already available, we use composite Gauß type quadrature rules, see e.g., [11].

### Numerical stability

Up to now, it has been shown how to derive an immersed finite element method for boundary value and interface problems, see (9) and (19), and how to compute the matrix coefficients of the linear system of equations. But the stable solution of this final system of Eq. (21) remains to be discussed, especially in view of the method’s parameters $\gamma$ (for boundary value and interface problems) and $\beta$ (for interface problems only).

#### Sources of instability

**Fig. 9:**
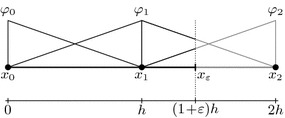
One-dimensional test example. Two linear finite elements with the right boundary inside the second element

As an illustrative example, consider a one-dimensional problem

27 $$\begin{aligned} - \alpha u^{\prime \prime }= & {} f \qquad x \in (0,x_\varepsilon )\nonumber \\ u= & {} 0 \qquad x = 0 \\ \alpha u^\prime = 0 \quad \text {or} \quad u= & {} 0 \qquad x = x_\varepsilon \nonumber \end{aligned}$$

for the domain $\Omega = (0,x_\varepsilon )$ and a constant material parameter $\alpha$. The boundary conditions are a prescribed value of $u=0$ at the left end and either a zero derivative (homogeneous Neumann) or a zero function value (homogeneous Dirichlet) at the right end. Let $\Omega _\square = (0,2h)$ be the embedding domain and two linear finite elements of size *h* are used for the discretisation, see Fig. 9. First, we consider the case with a homogeneous Neumann boundary condition at the right end. The left-side boundary condition is going to be incorporated essentially and the system matrix becomes

28 $$\begin{aligned} \varvec{\mathsf {K}}_N = \frac{\alpha }{h} \begin{pmatrix} 1+\varepsilon \quad -\varepsilon \\ -\varepsilon \qquad \varepsilon \end{pmatrix}\,. \end{aligned}$$

Obviously, for $\varepsilon \rightarrow 1$ this matrix recovers the standard finite element matrix for this problem with its known properties. The eigenvalues of this matrix have the values

29 $$\begin{aligned} \lambda _{1,2} = \frac{\alpha }{2h} \left[ 1 + 2\varepsilon \pm \sqrt{4 \varepsilon ^2 + 1} \right] \,. \end{aligned}$$

Clearly, the smaller eigevalue goes to zero for the limit $\varepsilon \rightarrow 0$, that is the case of a vanishing cut element. As expected, the matrix $\varvec{\mathsf {K}}_N$ is ill-conditioned for this limit.

We now turn to the Dirichlet case and evaluate the left-hand-side of expression (7) for this simple test problem. The resulting stiffness matrix has the form (replacing the surface integrals by point evaluation at $x_\varepsilon$)

30 $$\begin{aligned} \frac{h}{\alpha } \varvec{\mathsf {K}}_D = \begin{pmatrix} 1+\varepsilon \quad -\varepsilon \\ -\varepsilon \qquad \varepsilon \end{pmatrix} - \begin{pmatrix} \varepsilon -1 \quad 1-\varepsilon \\ -\varepsilon \qquad \varepsilon \end{pmatrix} - \begin{pmatrix} \varepsilon -1 \quad -\varepsilon \\ 1-\varepsilon \qquad \varepsilon \end{pmatrix} \nonumber \\ \;\,+\, \gamma \frac{h}{\alpha } \begin{pmatrix} (1-\varepsilon )^2 \quad \varepsilon (1-\varepsilon ) \\ \varepsilon (1-\varepsilon ) \qquad \varepsilon ^2 \end{pmatrix}\,. \end{aligned}$$

Note that the expression for the system matrix has been multiplied by the factor $\frac{h}{\alpha }$. The expressions of the eigenvalues of $\varvec{\mathsf {K}}_D$ are not easily determined, but the condition $\det (\varvec{\mathsf {K}}_D) > 0$ is more workable. Note that since the trace of the matrix is positive and equals $\lambda _1 + \lambda _2$, the condition of a positive determinant (recall $\det (\varvec{\mathsf {K}}_D) = \lambda _1 \lambda _2$) is sufficient for positive definiteness. One gets

31 $$\begin{aligned} \det (\varvec{\mathsf {K}}_D) = \frac{1}{h^2} \left( \frac{\varepsilon \gamma h}{\alpha } -1 \right) (1 + \varepsilon )> 0 \quad \Rightarrow \quad \gamma > \frac{\alpha }{h \varepsilon }\,. \end{aligned}$$

Fulfilment of this condition guarantees that the matrix is positive definite for a fixed mesh size *h*, but unfortunately it implies $\gamma \rightarrow \infty$ for $\varepsilon \rightarrow 0$. The use of a very large value for $\gamma$ can lead to undesired numerical problems.

In the case of the interface problems and formulation (19), the situation is slightly better. The extra parameter $\beta$ can be adjusted in a smart way such that a finite value of $\gamma$ is always achievable. Such a choice is proposed in [43] where $\beta$ depends on the material parameters of the subdomains and the sizes of the cut elements, $|\tau _I \cap \Omega _i|$. Using this approach, the system matrix has always positive eigenvalues (for the considered problem class) with a finite value of $\gamma$. Nevertheless, the minimal eigenvalue goes to zero for vanishing sizes of the cut elements. Even though the parameter choices by [43] show a good performance in terms of the quality of the numerical results, the matrix condition number still cannot be bounded for a fixed mesh and arbitrary interface locations.

#### Stabilisation

The above indicated sources of numerical instability all stem from the same situation that for some degrees of freedom, the intersection of the support of their associated shape functions with the physical domain becomes very small,

32 $$\begin{aligned} s_I = | {{\mathrm{supp}}}(\varphi _I) \cap \Omega | \ll h\,, \end{aligned}$$

where ${{\mathrm{supp}}}(\varphi _I)$ denotes the support of shape function $\varphi _I$ and *h* is a measure of the mesh size on $\Omega _\square$. In all above cases, Neumann, Dirichlet, or interface problem, this leads to severe ill-conditioning of the final system matrix. To solve this problem, the following approaches have been proposed, among others,

1. Discarding all degrees of freedom with support intersection below a certain threshold, $s_I < \varepsilon h$;
2. Adding a face-based stabilisation term [16];
3. Constraining degenerate degrees of freedom [6, 12].

As reported, among others, in [12], the approach S-1 leads to a loss of approximation order. Although appealing due to its simplicity, this drawback can be prohibitive in some applications. An ad-hoc approach to remedy the stability problem is to locally adapt the finite element mesh in order to avoid the problem of too small values of $s_I$. Even though simple at first sight, a robust realisation of this idea in three dimensions is not straightforward and mesh entanglement needs to be avoided. The support size $s_I$ is increased if specific nodes are moved away from the surface $\Gamma$, but there is an interesting alternative in which the points are snapped to the surface thereby generating a conforming mesh, see [44] for two-dimensional analysis of this idea.

Another approach, S-2, is proposed in [16] where the jump of the function gradients across certain element faces is added to the weak form in order to guarantee stability of the method. Other than the result (31), the system matrix stays well-conditioned for small values of $\gamma$ in the limit $\varepsilon \rightarrow 0$. This approach requires to evaluate surface integrals over interior mesh faces, a technicality which requires additional data structures in many codes, but does not add much to the overall difficulty of implementing an immersed finite element method. Nevertheless there is a drawback with this approach, since it introduces another weighting factor whose adjustment is not straightforward: for too small values of this factor the stabilisation effect disappears and for too large values the method’s accuracy is affected [16].

Finally, we consider S-3 which relies on the concept of coupling degrees of freedom with too small supports to other degrees of freedom from the interior of the domain. In order to outline this approach, the degrees of freedom shall first be classified according to the size $s_I$ of the intersection of their support with the domain, as defined in (32). In “Finite element discretisation” section, the set $\mathbb {S}$ has been introduced which contains all indices of shape functions for which $s_I$ is larger than zero. Introducing a threshold $\hat{s}$, the set $\mathbb {S}$ is now decomposed into the disjoint index sets, $\mathbb {A}$ and $\mathbb {B}$ with definition

33 $$\begin{aligned} \mathbb {A} = \{ I \in \mathbb {S} :s_I \ge \hat{s} \} \quad \text {and} \quad \mathbb {B} = \{ I \in \mathbb {S} :s_I < \hat{s} \}\,. \end{aligned}$$

The threshold $\hat{s}$ used in this classification has to depend on the mesh size *h* and should not be larger than one typical element size. The basic idea of Höllig et al. [12] is to constrain degrees of freedom from the set $\mathbb {B}$ to suitably chosen degrees of freedom from $\mathbb {A}(J)$, a subset of $\mathbb {A}$,

34 $$\begin{aligned} \forall J \in \mathbb {B}:\quad \varvec{u}_J = \sum _{I \in \mathbb {A}(J) \subset \mathbb {A}} c_{IJ} \varvec{u}_I \end{aligned}$$

where the coefficients $c_{IJ}$ will be discussed further below. These constraints give rise to the modified shape function basis

35 $$\begin{aligned} \varvec{u}^h(\varvec{x})= & {} \sum _{I\in \mathbb {A}} \varvec{u}_I \varphi _I(\varvec{x}) + \sum _{J\in \mathbb {B}} \varvec{u}_J \varphi _J(\varvec{x}) \nonumber \\= & {} \sum _{I\in \mathbb {A}} \varvec{u}_I \varphi _I(\varvec{x}) + \sum _{J\in \mathbb {B}} \left( \sum _{K \in \mathbb {A}(J)} c_{KJ} \varvec{u}_K \right) \varphi _J(\varvec{x}) \nonumber \\= & {} \sum _{I \in \mathbb {A}} \varvec{u}_I \left( \varphi _I(\varvec{x}) + \sum _{J \in \mathbb {B}(I)} c_{IJ} \varphi _J(\varvec{x}) \right) = \sum _{I \in \mathbb {A}} \varvec{u}_I \tilde{\varphi }_I(\varvec{x})\,. \end{aligned}$$

In this reordering of the finite element approximation (20) a new set $\mathbb {B}(I)$ is used which contains all indices *J* from $\mathbb {B}$, such that $I \in \mathbb {A}(J)$. For the implementation of this stabilisation method, it is sufficient to work with expression (34), but the result of (35) demonstrates that effectively a modified shape function basis $\{ \tilde{\varphi }_I\}_{I\in \mathbb {A}}$ is generated and illustrates the notion of *extended splines* as given in [12]. Note also that $\mathbb {B}(I) = \emptyset$ for all degrees of freedom that are not in the vicinity of the boundary and in that case $\tilde{\varphi }_I = \varphi _I$, so that most shape functions are not affected. Since the support size of the basis functions $\tilde{\varphi }_I$ is larger, the bandwidth increases for these degrees of freedom. Therefore, it has to be remarked that only degrees of freedom in the vicinity of the boundary are affected. Moreover, the storage requirement of the final system matrix is of course not larger than it would be for the original (unstable) basis functions $\varphi _I$.

**Fig. 10:**
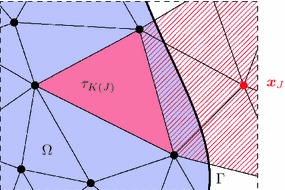
Cut-element stabilisation on a triangular mesh. Node $\varvec{x}_J$ is the location a degree of freedom $\varvec{u}_J$, $J \in \mathbb {B}$ and the element $\tau _{K(J)}$ is used for constraining $\varvec{u}_J$

There are two open questions when using this approach: (i) the choice of the index set $\mathbb {A}(J)$ associated to *J* and (ii) the values of the constraint weights $c_{IJ}$. The origin of this approach, as introduced in [12], is to stabilise b-spline discretisations. In this particular situation, the underlying mesh is logically Cartesian and an explicit expression of the coefficients $c_{IJ}$ can be given as a function of the multi-indices used to label that grid. See also [6] for a more intuitive interpretation of the arising extrapolation of Lagrange polynomials and its efficient implementation. The aim of this stabilisation procedure is to maintain the convergence order of the method and therefore to not lose the polynomial approximation quality of the approximation (20) due to the constraints (34). In other words, the modified basis functions $\tilde{\varphi }_I$ introduced in (35) have to represent the same polynomials as the $\varphi _I$ themselves.

In order to outline the procedure for obtaining $\mathbb {A}(J)$ and the corresponding coefficients $c_{IJ}$, consider the situation depicted in Fig. 10. The degree of freedom $\varvec{u}_J$, $J \in \mathbb {B}$, resides at node $\varvec{x}_J$ and the size of the intersection of the support (hatched in the picture) with the domain $\Omega$ is below the threshold $\hat{s}$. Searching through the elements in the vicinity of $\varvec{x}_J$, one finds the element $\tau _{K(J)}$ whose connected degrees of freedom all belong to $\mathbb {A}$. Any element entirely inside the domain $\Omega$ fulfils this condition. Normally, many such elements can be found and the closest is selected, where the distance between the element middle point and $\varvec{x}_J$ is a possible way to measure the proximity. The selected element $\tau _{K(J)}$ gives rise to the index set $\mathbb {A}(J) \subset \mathbb {A}$ associated with $\varvec{u}_J$. Formally, we can write

36 $$\begin{aligned} \mathbb {A}(J) = \{ I \in \mathbb {A} :{{\mathrm{supp}}}(\varphi _I) \cap \tau _{K(J)} \ne \emptyset \}\,. \end{aligned}$$

Once this set is defined, the weights $c_{IJ}$ are calculated by evaluation of the basis of $\tau _{K(J)}$ at the node $\varvec{x}_I$,

37 $$\begin{aligned} \forall I \in \mathbb {A}(J):c_{IJ} = \varphi _I(\varvec{x}_J). \end{aligned}$$

This choice of weights is an extension of the idea given in [15] where the weights are defined for non-uniform b-splines as dual functionals applied to the polynomials in a chosen grid element. Here the point evaluation of (37) is the corresponding dual functional of Lagrange polynomials [8]. Note that $\varvec{x}_J \notin \tau _{K(J)}$ and thus $c_{IJ}$ represents an extrapolation of the polynomial basis spanned in $\tau _{K(J)}$ to the outside point $\varvec{x}_J$, see also Fig. 10. The stabilisation procedure can be summarised as follows

1. categorise $\mathbb {A}$ and $\mathbb {B}$ using a threshold $\hat{s}$, see (32) and (33)
2. for all $$J \in \mathbb {B}$$
  find $\tau _{K(J)}$ with all degrees of freedom from $\mathbb {A}$ that is close to $\varvec{x}_J$,
  define the constraint coefficients as $c_{IJ} = \varphi _I(\varvec{x}_J)$ for all $I \in \mathbb {A}(J)$
3. assemble the final system of equations using the constraint equations (34) applied to test and trial spaces
4. after solving the global system, calculate the constrained degree of freedom $\varvec{u}_J$ with $J \in \mathbb {B}$ according to (34).

**Fig. 11:**
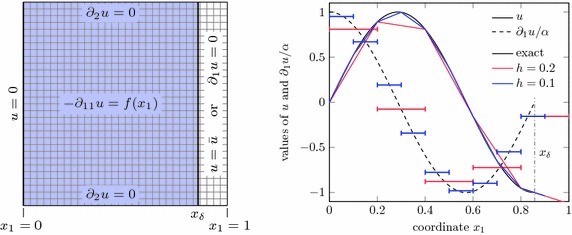
One-dimensional problem. Setup (*left*) and a comparison of exact with approximate solution and its derivative for $x_\delta =\frac{6}{7}$ (*right*)

With respect to the implementation a few remarks have to be made. The code has to be able to search the elements in the neighbourhood of a given node. For instance, the element $\tau _{K(J)}$ in Fig. 10 does not lie in the support of $\varphi _J$ but in the ring of elements around that support. Theoretically, for very extreme shapes of $\Gamma$ the nearest $\tau _{K(J)}$ to $\varvec{x}_J$ could lie far away, but here we assume that the mesh is fine enough such that there is always an element nearby. Cusp-shaped domains are excluded from the onset. In addition, one has to evaluate the shape functions of $\tau _{K(J)}$ at $\varvec{x}_J$ and this requires to find first the reference coordinate $\varvec{\xi }_J$ (outside of the reference element) such that the geometry representation of the chosen element represents $\varvec{x}_J$ when evaluated at this coordinate, that is $\varvec{x}_{K(J)} (\varvec{\xi }_J) = \varvec{x}_J$. Here we restrict ourselves to meshes in which all elements are an affine transformation of the reference element. Higher-order geometry representations of the volume mesh are excluded, but they are also not necessary since the mesh, by design of the immersed method, need not conform to the geometry of $\Omega$.

## Numerical examples

At last, a few numerical examples are presented in order to study and demonstrate the performance of the immersed finite element method as presented here. Unless indicated otherwise, the spatial discretisation of all problems is carried out with linear finite elements. As shown in the appendix, the Nitsche parameter is chosen as $\gamma = \gamma _0 \tfrac{\alpha }{h}$ with the mesh width *h*, the representative material parameter $\alpha$ and a dimensionless scalar $\gamma _0$. The default choices for this parameter is $\gamma _0 = 10$ and for interface problems the additional parameter is chosen as $\beta = 0.5$. The threshold $\hat{s}$ used to distinguish between the degree of freedom sets $\mathbb {A}$ and $\mathbb {B}$ in the stabilisation of “Stabilisation” section is set to the size of one element.

### Convergence and robustness analysis

At first, the method’s performance under variation of various parameters is assessed. For this purpose, an essentially one-dimensional Poisson problem is used as depicted in Fig. 11, left, with a forcing function $f(\varvec{x}) = \alpha ^2 \sin (\alpha x_1)$ and $\alpha = \frac{3\pi }{2 x_\delta }$. The resulting exact solution is then $u(\varvec{x}) = \sin (\alpha x_1)$. The signed distance function is ${{\mathrm{dist}}}_\Gamma (\varvec{x}) = x_\delta - x_1$ and the boundary is represented exactly. At first, this problem is analysed using a structured mesh as shown in the figure. Figure 11, on the right, shows the analytic solution (solid black line) along the $x_1$-axis and its derivative (dashed line) for a boundary location at $x_\delta = \frac{6}{7}$. The approximations $u^h$ and $\partial _{1} u^h$ for a mesh with $5\times 5$ elements (red) and a $10\times 10$ element (blue) are also displayed. One can see that the numerical approximation $u^h$ coincides with the analytic solution at the finite element nodes and, moreover, at the boundary location at $x_\delta$.

**Fig. 12:**
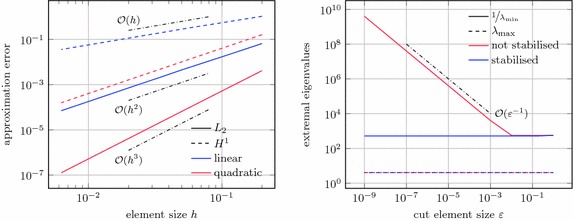
Neumann problem. Convergence for $x_\delta = \frac{6}{7}$ (*left*) and smallest and largest matrix eigenvalues for a fixed mesh width $h=0.025$ and various boundary locations $x_\delta = (34+\varepsilon )h$

The convergence of the method is shown in the left of Fig. 12, where the numerical errors in $L_2$-norm and $H^1$-seminorm are shown for an approximation with linear and a quadratic Lagrange polynomials. These results exhibit the expected optimal convergence rates [8]. In this graph, the boundary location is held fixed at $\varvec{x}_\delta = \frac{6}{7}$ and the mesh width *h* is decreased. On the other hand, the right side of Fig. 12 shows the smallest and largest eigenvalues of the system matrix in dependence of the boundary location for a non-stabilised implementation and for the stabilisation presented in “Stabilisation” section. Here, a fixed $40\times 40$ mesh is used and the location of the boundary is at $\varvec{x}_\delta = (34+\varepsilon )h$ with the parameter $0 \le \varepsilon \le 1$. A Neumann boundary condition at $\varvec{x}_\delta$ is considered and, hence, one has always $a(u^h,u^h) > 0$ and $\lambda _{\min } > 0$. One can clearly see that $\lambda _{\min } \in \mathcal {O}(\varepsilon )$ for small $\varepsilon$ and for the non-stabilised case (note that the figure shows in fact the inverse $\frac{1}{\lambda _{\min }}$). Clearly, the matrix condition number grows without bound. The stabilisation as outlined in “Stabilisation” section, however, guarantees a constant value of $\lambda _{\min }$ well above zero. The largest eigenvalues coincide for both cases.

**Fig. 13:**
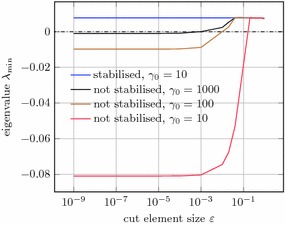
Dirichlet problem. Smallest eigenvalue for various boundary locations ($h=0.025$ and $x_\delta = (34+\varepsilon )h$)

Now we turn to the problem with a Dirichlet boundary condition at $x_\delta$. Using the same variation of the location of this boundary as above, Fig. 13 shows the smallest eigenvalue $\lambda _{\min }$ for the stabilised method and for the non-stabilised method for various values of $\gamma _0$ (recall that $\gamma = \frac{\gamma _0}{h}$). One can see that without stabilisation the considered minimal eigenvalue changes sign for decreasing values of $\varepsilon$ rendering the system matrix indefinite (and singular when the zero is crossed). In order to force $\lambda _{\min } >0$ one can increase the value of $\gamma _0$, but for $\varepsilon \rightarrow 0$ this value grows without bound and one gets effectively $\lambda _{\max } \rightarrow \infty$ which likewise deteriorates the condition number of the matrix, as already discussed in “Sources of instability” section.

**Fig. 14:**
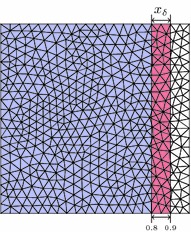
Unstructured mesh. Varying location of the right boundary at $x_\delta$

**Fig. 15:**
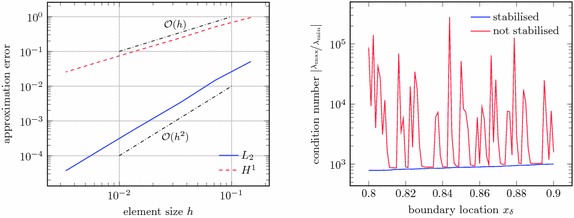
Results for unstructured mesh. Convergence of the Neumann problem on the unstructured mesh for fixed $x_\delta = \frac{6}{7}$ (*left*) and the matrix condition number for various boundary locations $0.8 \le x_\delta \le 0.9$ (*right*)

Next, the stabilisation technique is applied to an unstructured mesh as shown in Fig. 14. Note that this case is not covered by the original idea of this technique as given by [12] which was only designed for b-spline basis functions on structured meshes. The left of Fig. 15 shows the convergence of the stabilised method for linear triangle elements and a fixed boundary location. Finally, the location of the boundary is varied again and the condition number for a Neumann problem is considered in the right of Fig. 15. Whereas in the non-stabilised case this value shows a very erratic behaviour with large peaks, the condition number for the stabilised method is almost constant at a low value.

**Fig. 16:**
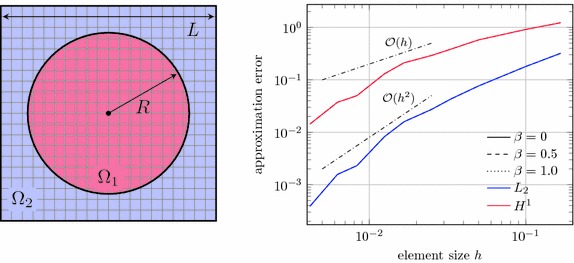
Interface problem. Computational setup of a square with a circular inclusion (*left*), convergence behaviour for different interface weights $\beta$ (*right*); note that these curves are not distinguishable

Now, an interface problem is considered. Figure 16 shows the computational domain that is composed of a circular domain $\Omega _1$ embedded in a square domain $\Omega _2$. On this domain, the Poisson problem $- \alpha _i \Delta u = 4$ with material parameters $\alpha _1 = 1$ and $\alpha _2 = 1000$ is solved, subject to Dirichlet boundary conditions on the outer boundary $\partial \Omega _2$. The geometric parameters are chosen as $R=0.75$ and $L=2$, respectively. This problem together with its analytic solution is taken from [2]. In the right graph of Fig. 16 the convergence behaviour is shown for different values of the interface weight factor $\beta$. For the three considered values $\beta = 0$, 0.5 and 1, the curves are indistinguishable. Also optimal convergence rates are achieved for mesh sizes smaller than $h \approx 0.02$.

**Fig. 17:**
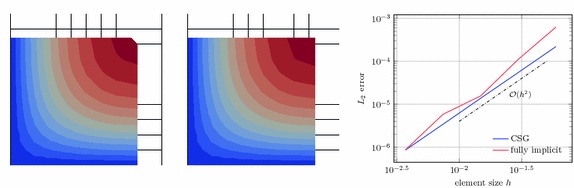
Influence of geometry representation. Implicit representations of a square (*left*: fully implicit, *middle*: mesh-based CSG) together with the *contour colours* of the solution to a Poisson equation, convergence of $L_2$ error (*right*)

At last, we consider the influence of the geometry representation. As outlined in “Constructive solid geometry modelling” section, we have to approaches available: the use of a signed distance function representing the entire embedded surface and the successive embedding of the geometry primitives that form the final model. For simplicity, consider a square that coincides on two of its edges with the mesh boundary whereas the other two are represented implicitly. Figure 17 shows the effect of the introduced two approaches in the left and middle images, respectively. Clearly, the upper right corner is chamfered off in the first approach, but represented exactly in the second. As a numerical problem we have chosen $-\Delta u = 1$ on a unit square subject to $u=0$ on the lower and left boundaries and $\partial u / \partial \varvec{n} = 0$ on the other two boundaries. An analytic solution to this problem is available, for instance in [45] in the context of Poiseuille flow in a rectangular channel. The right graph in Fig. 17 shows the convergence rates for the two types of geometry modelling. Clearly, optimal convergence rates are obtained for both cases. Nevertheless the exact representation of the corner leads to a much smoother outcome with lower approximation errors for coarse mesh sizes.

### Mesh-embedded CSG

**Fig. 18:**
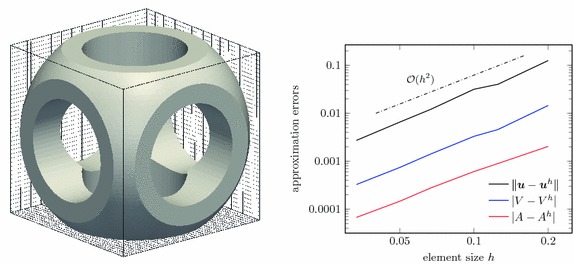
CSG modelling. Embedded domain (*left*) and convergence behaviour of the displacement $\varvec{u}$, the domain’s volume *V* and surface area *A*

Here the domain as obtained by the CSG process of Fig. 6 is reconsidered, see also the left of Fig. 18. The embedding domain is $\Omega _\square = (0,1)^3$ and equipped with a uniform hexahedral mesh. Following the mesh-based Boolean operations as introduced in “Constructive solid geometry modelling” section, the immersed geometry is obtained by

1. *intersection* with a sphere of radius 0.65 and centred at (0.5, 0.5, 0.5), and
2. successive *subtraction* of cylinders around the same centre with radius 0.3 and in the directions of the $x_i$-coordinate axes.

Thus the domain $\Omega$ is obtained as shown in Fig. 18 and we assume that it is occupied by a hyperelastic solid. In a first analysis, linearised elasticity is assumed and the convergence is studied by using fundamental solution of elasticity $\varvec{U}(\varvec{x},\varvec{y})$ (see, for instance, [46]) as an imposed analytic solution with a source point $\varvec{y}$ located outside of the domain. Therefore, on the bottom ($x_3=0$) the boundary displacements $\bar{\varvec{u}}(\varvec{x}) = \varvec{U}(\varvec{x},\varvec{y})$ are prescribed and the remaining boundaries are subject to the Neumann condition $\bar{\varvec{t}} = \varvec{t}(\varvec{U})(\varvec{x},\varvec{y})$. For simplicity, the material parameters are chosen as $\lambda = 28.85$ and $\mu = 19.23$. The right of Fig. 18 shows the convergence of the displacement solution and of the computed volume and surface area of the embedded domain. Quadratic convergence is observed for all considered quantities.

**Fig. 19:**
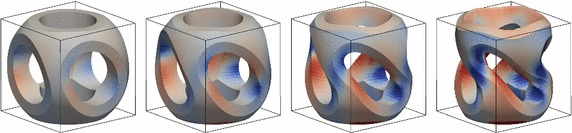
Large elastic deformation. Load steps 1–4; the surface is *coloured* by the stress component $S_{33}$

Next, a compressible Neo-Hookean material model [22, 23] with large deformations is used, based on the strain energy density

38 $$\begin{aligned} W(\varvec{F}) = \frac{\lambda }{2} (\log J)^2 - \mu \log J + \frac{\mu }{2} ({{\mathrm{tr}}}\varvec{C} - 3 )\,, \quad J = \det \varvec{F} \quad \text {and} \quad \varvec{C} = \varvec{F}^\top \varvec{F}, \end{aligned}$$

with the deformation gradient $\varvec{F} = \varvec{I} + {{\mathrm{Grad}}}\varvec{u}$. The material parameters are the same as in the linearised case above. For the example, the bottom boundary is held fixed and a twisting traction field is applied to the top surface with value $\bar{\varvec{t}} = 10 ( x_2 - 0.5, 0.5 - x_1, 0)$. The load is applied in 4 steps and within each step a Newton method is used to obtain the equilibrium state. The deformed geometry for these four load steps is shown in the images of Fig. 19 for a $40^3$ grid of linear hexahedron elements.

**Fig. 20:**
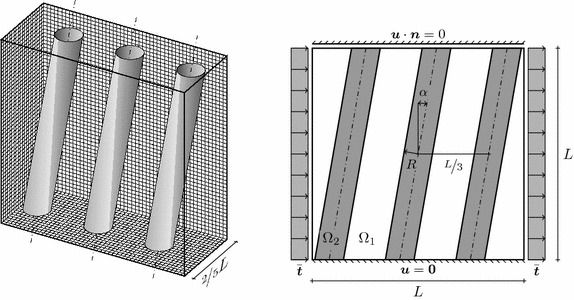
Fibre-reinforced material. Three-dimensional view (*left*) and front view (*right*); in this drawing $\alpha =10^\circ$

### Composite material

As a last example, the elastic deformation of a fibre-reinforced block of elastic material is considered. A block of dimension $L \times \frac{2}{5} L \times L$ is reinforced by inclined fibres placed with a main axis separation of $\frac{L}{3}$. The fibres are represented by cylinders with radius $\frac{L}{15}$. The three-dimensional setup is shown in the left of Fig. 20 and on the right a two-dimensional view of the problem is depicted. The bottom surface is held fixed and the top surface is constrained in normal direction. The left and right surfaces are subject to a constant traction field $\bar{\varvec{t}}$ in normal direction. The discretisation is carried out by a fixed mesh of dimension $50 \times 20 \times 50$ as indicated on the back faces of the three-dimensional view. For comparison, we monitor the average horizontal displacement

39 $$\begin{aligned} U_1 = \frac{1}{|\Omega |} \int _\Omega u_1(\varvec{x}) \mathrm {d}\Omega \end{aligned}$$

throughout the composite body for a variety of fibre angles $-35^\circ \le \alpha \le 35^\circ$. Both domains $\Omega _1$ and $\Omega _2$ have the hyperelastic material law according to the energy (38) and the computations are carried out with large deformations. For comparison, a linearised situation is also considered.

**Fig. 21:**
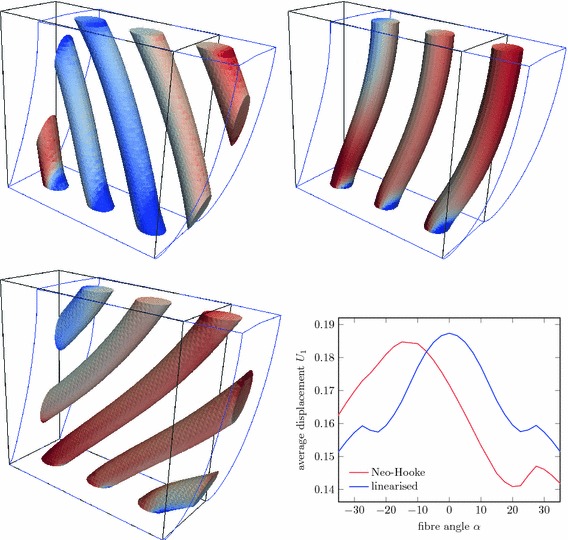
Computational analysis of fibre-reinforced material. Deformed geometry with fibres coloured by $S_{33}$ for $\alpha =-35^\circ$ (*top left*), $\alpha = 0^\circ$ (*top right*), and $\alpha =+35^\circ$ (*bottom left*); average horizontal displacement $U_1$ for various fibre angles (*bottom right*)

The model parameters are chosen as $L=1$ and $\bar{\varvec{t}}=(1,0,0)$. The materials are represented by the Lamé parameters $\lambda _1 = 5.769$, $\mu _1 = 3.846$, $\lambda _2 = 10 \lambda _1$ and $\mu _2 = 10 \mu _1$. Figure 21 shows the deformed configuration for fibre angles $\alpha =-35^\circ$, $\alpha =0^\circ$ and $\alpha =+35^\circ$. In addition, the analysis of the average horizontal displacement $U_1$ as a function of the considered fibre angles $\alpha$ is shown for the Neo-Hooke material and linearised elasticity. Although there are similarities between the large-deformation analysis and the linearised version, striking differences can be observed too. Most of all, the linear variant is completely symmetric with respect to the sign of $\alpha$ and has its largest value for $\alpha =0^\circ$. In the large-deformation variant, on the other hand the result is a larger deformation for the negative fibre angles and smaller for positive angles. Overall, the body behaves less flexibly in the nonlinear analysis, but with a strong bias to an increased flexibility for negative fibre angles.

## Conclusions

Immersed finite element methods, that do not rely on a body-fitted mesh, are a promising alternative to conventional FEM for many applications. Especially in the case of complex three-dimensional geometries, moving interfaces, or design optimisation such methods allow for more flexible geometry processing and remove the repeated interaction with mesh generation software. Here, we present an immersed FEM for the problem class of nonlinear elasticity, based on a weak incorporation of Dirichlet boundary conditions and interface conditions with Nitsche’s method, an implicit geometry representation and accurate integration of the arising cut elements. We place emphasis on the implementation details such as the robust computation of the signed distance function and quadrature by means of tessellation. A common pitfall of non-body-fitted FEM, the loss of numerical stability in situations with degenerate function support, is analysed and we provide a stabilisation technique that is robust without affecting the convergence behaviour. Moreover, the choice of the parameters in the context of Nitsche’s method are thoroughly discussed.

We demonstrate a way to incorporate sharp features such as edges and vertices in our method by means of successively embedding the geometry primitives into the analysis mesh in a similar way as in constructive solid geometry modelling. Based on this idea, geometry modelling is directly integrated in the finite element analysis and there is no need for a mesh generation tool. The presented applications emphasise the potential of this approach, where large deformation analyses are carried out based on a trivial Cartesian background mesh.

A present shortcoming of the introduced approach is the restriction to linear approximation orders. Although the field approximation used in this FEM can be of arbitrary order, a gain in convergence order would be impeded by the geometry representation based on linear facets. In principle, the use of more accurate signed distance functions and the subsequent adaptation on the quadrature level to account for embedded higher-order surface representations is feasible.

Finally, we note that the presented method is ideally suited for the incorporation of *h*-adaptivity. A combination of this immersed FEM with hierarchical refinement techniques as shown, for instance, in [47] would render a powerful analysis toolbox, which yields accurate numerical predictions based only on the input of geometry primitives.

## Appendix A: method parameters $\gamma$ and $\beta$

In the following, estimates for the parameters $\gamma$ (Penalty weighting parameter first appearing in (8)) and $\beta$ (interface weighting parameter first appearing in (13)) are derived. These parameters only make sense with the Finite Element discretisation as the final goal. With a slight abuse of notation, the functions used in the following have to be understood as discrete FE solutions, but for simplicity the superscripts, for instance of $\varvec{u}^h$, have been omitted.

### Dirichlet boundary conditions

We develop an estimate for the parameter $\gamma$ appearing in the linearised weighted residual equation (9). The material behaviour is assumed to be such that $\mathrm {D}a(\varvec{u},\varvec{v})[\Delta \varvec{u}]$ is an elliptic bilinear form. The aim is to show that $A(\varvec{u};\varvec{w},\varvec{w}) > 0$ for any $\varvec{w}\ne \varvec{0}$, where $\varvec{u}$ is the current displacement solution. This gives

40 $$\begin{aligned} A(\varvec{u};\varvec{w},\varvec{w})= & {} \mathrm {D}a(\varvec{u},\varvec{w})[\varvec{w}] - 2 \int _{\Gamma _D} \mathrm {D}\varvec{t}(\varvec{u})[\varvec{w}] \cdot \varvec{w}\mathrm {d}\Gamma + \gamma \int _{\Gamma _D} \varvec{w}\cdot \varvec{w}\mathrm {d}\Gamma \nonumber \\\ge & {} \mathrm {D}a(\varvec{u},\varvec{w})[\varvec{w}] - 2 \Vert \mathrm {D}\varvec{t}(\varvec{u})[\varvec{w}] \Vert _{\Gamma _D} \Vert \varvec{w}\Vert _{\Gamma _D} + \gamma \Vert \varvec{w}\Vert _{\Gamma _D}^2 \nonumber \\\ge & {} \mathrm {D}a(\varvec{u},\varvec{w})[\varvec{w}] - 2 C \sqrt{\mathrm {D}a(\varvec{u},\varvec{w})[\varvec{w}]} \Vert \varvec{w}\Vert _{\Gamma _D} + \gamma \Vert \varvec{w}\Vert _{\Gamma _D}^2 \nonumber \\= & {} \left( \sqrt{\mathrm {D}a(\varvec{u},\varvec{w})[\varvec{w}]} - C \Vert \varvec{w}\Vert _{\Gamma _D} \right) ^2 +(\gamma - C^2) \Vert \varvec{w}\Vert _{\Gamma _D}^2 \,. \end{aligned}$$

Here, $\Vert \varvec{w}\Vert _{\Gamma _D}$ is the $L_2$-norm over the Dirichlet boundary $\Gamma _D$. The Cauchy-Schwarz inequality has been used from the first to the second line and, most importantly, the third line is based on the inverse inequality

41 $$\begin{aligned} C^2 \mathrm {D}a(\varvec{u},\varvec{w})[\varvec{w}] \ge \Vert \mathrm {D}\varvec{t}(\varvec{u})[\varvec{w}] \Vert _{\Gamma _D}^2\,. \end{aligned}$$

This type of estimate is also presented in [3] for the case of Poisson’s equation. Knowledge of the constant *C* gives rise to the choice $\gamma > C^2$ which renders the last line in (40) positive. Inserting the finite element trial functions (20) into (41) leads to the condition

42 $$\begin{aligned} C^2 \mathrm {D}a(\varvec{u}, \varvec{e}_a \varphi _I)[ \varvec{e}_b \varphi _J ] \ge \int _{\Gamma _D} \mathrm {D}\varvec{t}(\varvec{u})[\varvec{e}_a \varphi _I] \cdot \mathrm {D}\varvec{t}(\varvec{u})[\varvec{e}_b \varphi _J] \mathrm {d}\Gamma \end{aligned}$$

for all coordinate directions $\varvec{e}_a$, $0 \le a < n_d$, and all shape functions $\varphi _I$. Obviously, this condition is only non-trivial if the supports of $\varphi _I$ and $\varphi _J$ overlap each other and intersect with the Dirichlet boundary $\Gamma _D$. Therefore, we use the following abbreviations for these domain and boundary intersections

43 $$\begin{aligned} \Omega _{IJ} = \Omega \cap ({{\mathrm{supp}}}(\varphi _I) \cap {{\mathrm{supp}}}(\varphi _J)) \quad \text {and} \quad \Gamma _{IJ} = \Gamma _D \cap ({{\mathrm{supp}}}(\varphi _I) \cap {{\mathrm{supp}}}(\varphi _J)). \end{aligned}$$

The left-hand side of (42) can be re-written as

44 $$\begin{aligned} \mathrm {D}a(\varvec{u},\varvec{e}_a \varphi _I)[\varvec{e}_b \varphi _J] = \int _{\Omega _{IJ}} \varvec{{{\mathrm{Grad}}}}(\varvec{e}_b \varphi _J) :\hat{\varvec{C}}(\varvec{u}) :\varvec{{{\mathrm{Grad}}}}(\varvec{e}_a \varphi _I) \mathrm {d}\Omega \,, \end{aligned}$$

and the right-hand side becomes

45 $$\begin{aligned}&\int _{\Gamma _{IJ}} \left[ \left( \hat{\varvec{C}}(\varvec{u}) :\varvec{{{\mathrm{Grad}}}} (\varvec{e_a} \varphi _I) \right) \varvec{n} \right] \cdot \left[ \left( \hat{\varvec{C}}(\varvec{u}) :\varvec{{{\mathrm{Grad}}}} (\varvec{e_b} \varphi _J) \right) \varvec{n} \right] \mathrm {d}\Gamma \nonumber \\&\quad =\int _{\Gamma _{IJ}} \left( \hat{\varvec{C}}(\varvec{u}) :\varvec{{{\mathrm{Grad}}}} (\varvec{e_a} \varphi _I) \right) \left( \varvec{n} \otimes \varvec{n} \right) :\left( \hat{\varvec{C}}(\varvec{u}) :\varvec{{{\mathrm{Grad}}}} (\varvec{e_b} \varphi _J) \right) ^\top \mathrm {d}\Gamma \nonumber \\&\quad \le \int _{\Gamma _{IJ}} \left( \hat{\varvec{C}}(\varvec{u}) :\varvec{{{\mathrm{Grad}}}} (\varvec{e_a} \varphi _I) \right) :\left( \hat{\varvec{C}}(\varvec{u}) :\varvec{{{\mathrm{Grad}}}} (\varvec{e_b} \varphi _J) \right) \mathrm {d}\Gamma \,. \end{aligned}$$

Abbreviating the integrands in (44) and (45) with $f^\Omega _{aIbJ}$ and $f^\Gamma _{aIbJ}$, we obtain for the inverse inequality

46 $$\begin{aligned} C^2 \int _{\Omega _{IJ}} f^\Omega _{aIbJ} \mathrm {d}\Omega \ge \int _{\Gamma _{IJ}} f^\Gamma _{aIbJ} \mathrm {d}\Gamma \,. \end{aligned}$$

Assuming continuity of the integrands, the mean value theorem of integration states that two points $\varvec{\xi } \in \Omega _{IJ}$ and $\varvec{\eta } \in \Gamma _{IJ}$ exist such that the estimate becomes

47 $$\begin{aligned} C^2 \ge \frac{f^\Gamma _{aIbJ}(\varvec{\eta })}{f^\Omega _{aIbJ} (\varvec{\xi })} \frac{|\Gamma _{IJ}|}{|\Omega _{IJ}|}\,. \end{aligned}$$

Without assumptions on the shape functions $\varphi _I$ and the material behaviour, we cannot further reduce the ratio of the evaluated integrands. Nevertheless, if $\alpha$ denotes a characteristic material behaviour (for instance the bulk modulus), it is clear from (44) and (45) that this ratio scales like $\frac{\alpha ^2}{\alpha } = \alpha$. Here, we focus on controlling the second part of the right hand side of the estimate (47). Using a standard finite element basis, there are constellations in which $\Omega _{IJ} \rightarrow 0$, whereas $\Gamma _{IJ}$ does not decrease: see, for instance the *sliver* test case in [16].

Employing the proposed stabilisation technique from “Stabilisation” section, where critical shape functions are essentially joined with neighbouring ones that have a non-degenerate support, the above geometric ratio can be safely estimated as

48 $$\begin{aligned} \frac{|\Gamma _{IJ}|}{|\Omega _{IJ}|} \approx \frac{h^{n_d-1}}{h^{n_d}} = \frac{1}{h}\,, \end{aligned}$$

because the support sizes of the stabilised basis functions $\tilde{\varphi }_I$ never fall below the stabilisation threshold $\hat{s}$ which is of order $h^{n_d}$. In conclusion, we can state that there exists a constant $\gamma _0$ independent of the material and the mesh size *h*, such that

49 $$\begin{aligned} \gamma = \gamma _0 \frac{\alpha }{h} > C^2 \end{aligned}$$

and, in turn, $A(\varvec{u}; \varvec{w},\varvec{w}) > 0$, see estimate (40).

### Interface conditions

Next, we assess the choice of parameters in the linearised weighted residual equation (19) for the interface problems. To this end, the steps of (40) are repeated in an analogous manner with the function $\varvec{w} = (\varvec{w}_1, \varvec{w}_2)$ and yield the estimate

50 $$\begin{aligned}&A(\varvec{u}; \varvec{w},\varvec{w})\nonumber \\&\quad \ge \left( \sqrt{ \mathrm {D}a_1(\varvec{u}_1,\varvec{w}_1)[\varvec{w}_1] + \mathrm {D}a_2(\varvec{u}_2,\varvec{w}_2)[\varvec{w}_2] } - D \Vert \llbracket \varvec{w} \rrbracket \Vert _\Gamma \right) ^2 + (\gamma - D^2) \Vert \llbracket \varvec{w} \rrbracket \Vert _\Gamma ^2\,,\nonumber \\ \end{aligned}$$

which is positive for $\varvec{w}_i \ne \varvec{0}$ and $\gamma > D^2$. Here, *D* is the constant of the inverse estimate

51 $$\begin{aligned}&D^2 \left( \mathrm {D}a_1(\varvec{u}_1, \varvec{w}_1)[\varvec{w}_1] + \mathrm {D}a_2(\varvec{u}_2,\varvec{w}_2)[\varvec{w}_2] \right) \nonumber \\&\quad \ge \int _{\Gamma } \left\{ \beta \mathrm {D}\varvec{t}(\varvec{u}_1)[\varvec{w}_1] + (1-\beta ) \mathrm {D}\varvec{t}(\varvec{u}_2)[\varvec{w}_2] \right\} ^2 \mathrm {d}\Gamma \,. \end{aligned}$$

Using the “Peter-Paul inequality” with some $\delta > 0$ (that is, the estimate $2ab \le \delta a^2 + \delta ^{-1} b^2$) one gets

52 $$\begin{aligned}&D^2 \left( \mathrm {D}a_1(\varvec{u}_1,\varvec{w}_1)[\varvec{w}_1] + \mathrm {D}a_2(\varvec{u}_2,\varvec{w}_2)[\varvec{w}_2] \right) \nonumber \\&\quad \ge (1+\delta ) \beta ^2 \int _{\Gamma } (\mathrm {D}\varvec{t}(\varvec{u}_1)[\varvec{w}_1])^2 \mathrm {d}\Gamma + (1+\delta ^{-1}) (1-\beta )^2 \int _{\Gamma } (\mathrm {D}\varvec{t}(\varvec{u}_1)[\varvec{w}_1])^2 \mathrm {d}\Gamma \,. \end{aligned}$$

Let $C_i$ denote the constant of (41) for the subdomain $\Omega _i$, choose $\delta = ((1-\beta )^2 C_2^2) / (\beta ^2 C_1^2)$, and insert the inverse inequality (41) into the last expression in order to get

53 $$\begin{aligned} D^2 \ge \beta ^2 C_1^2 + (1-\beta )^2 C_2^2 \,. \end{aligned}$$

Based on the discussion above we know that the stabilisation technique given in “Stabilisation” section keeps the values of $C_i$ bounded: therefore *D* according to (53) is well-behaved, independent of the choice of $0 \le \beta \le 1$. Nevertheless, in [43] a parameter choice for $\beta$ is presented which keeps the value of *D* bounded even for a non-stabilised finite element basis. Their choice is, adapted to the notation used here, $\beta = C_1^{-1} / (C_1^{-1} + C_2^{-1})$ and this value controls nicely the right-hand-side of expression (53) for unbounded values of $C_i$ (note that in case of geometric constellations for which $C_1$ is large the counterpart $C_2$ is small, and vice versa). But it has to be remarked that in certain types of applications (e.g. fluid-structure interaction [48]) it is convenient to freely choose the parameter $\beta$ without stability restrictions. Moreover, this special choice of $\beta$ maintains $A(\varvec{u}; \varvec{w}, \varvec{w}) >0$ for a finite value of $\gamma$, but does not prevent unbounded values of the condition number of the system matrix. With the here presented stabilisation technique, the choice of $\beta$ does not affect stability and $\gamma = \gamma _0 (\alpha _1 + \alpha _2) /h$ provides a safe choice for the characteristic material parameters $\alpha _i$.
